# Supplementary material for: Antimicrobial Resistance Policy Protagonists and Processes—A Qualitative Study of Policy Advocacy and Implementation
Source: Antibiotics (Basel). 2022 Oct 18;11(10):1434. doi: 10.3390/antibiotics11101434 (PMC9598113; doi:10.3390/antibiotics11101434)
Supplement: Supplementary file 1 [file antibiotics-11-01434-s001.zip › antibiotics-1916158-supplementary.pdf]

antibiotics

MDPI

Supplementary File

Table S1. Coding categorized as micro-determinants of AMR policymaking.

Level code	Sub-level code	Open coding - primary variables	Sub-variables-->	Axial Coding-->	Selective coding-->	MI with meso-construct-->	Policy protagonists in policy advocacy and implementation
MI	1	Perceived responsibility in personal advocacy/personal championship		Positive personal championship characteristics-significance of policy entrepreneurship in advocacy	Personal championship approaches	Personal championship and responsibility obligation	Personal obligation in personal championship
MI	2	Perceived personal political capacity and connections	2.1. Perceived connection with international peers on AMR issues 2.2. Perceived importance and willing to take personal risk	Positive personal championship characteristics particularly on politics nexus	Personal championship approaches	Personal championship and political connect	Political proximity in personal champsionship
MI	3	Perceived risk or benefits in political career	3.1. Lack of political benefit 3.2. Beneficial to political position 3.3. Internal: Perceived economic priority that helps political position 3.4. External: Helps build international common goal	Positive personal championship characteristics	Personal championship approaches	Personal championship and incentives vs dis-incentives in political career	Political incentives in personal champsionship
MI	4	Perception of sufficient support to advocate, prioritize or formulate policy		Positive personal championship characteristics particularly on politics nexus	Personal championship approaches	Personal championship and political connect	Political proximity in personal champsionship
MI	5	Of opinion that AMR is a traceable problem translatable to policy		Scientific evidence and knowledge channeling		Personal championship and personal belief	Personal belief in personal championship

**MI**	**6**	**Perception of positive possibility to prioritize AMR on agenda**	**6.1. Of opinion that AMR is a public health gap that needs to be addressed 6.2. Of opinion that AMR is an intellectual challenge**	**Positive personal championship characteristics**	**Personal championship approaches**	**Personal championship and policy prioritization**	**Policy prioritization**
**MI**	**7**	**Of opinion that AMR is a solvable issue**	**7.1. Of opinion that AMR can be a common goal among international country level peers 7.2. Of opinion that AMR is not solvable**	**Positive personal championship characteristics**	**Personal championship approaches**	**Personal championship and belief in solvability**	**Belief in solvability in personal championship**
**MI**	**8**	**Of opinion that AMR aligns with decision maker's core belief**	**8.1. Personal wish to expand AMR mitigation 8.2. /16 Vested personal in an established AMR theme/goal by the office or society or peer 8.3. Vested group of aligned interest to influence agenda prioritization 8.4. PM interest/agenda priority 8.5. PM's intellectual curiosity**	**Positive personal championship characteristics**	**Personal championship approaches**	**Personal championship and personal belief alignment**	**Personal belief in personal championship**
**MI**	**9**	**Of opinion that AMR is a priority within office or former office**		**Organization determinants**	**Organization determinants**	**Personal championship and view of office commitment**	**Office role commitment in personal championship**
**MI**	**10**	**Previous knowledge and experience influence belief in mitigating AMR**		**Positive personal championship characteristics**	**Personal championship approaches**	**Personal championship and belief in knowledge of**	**Knowledge-based incentive in personal championship**

					AMR mitigation	
MI	11	Group of advocates/network coming together for AMR cause	Organization determinants	Organization determinants	Institutional characteristics and advocacy coalition	Institutional-based incentive in personal championship
MI	12	Personal-ecostructure-advocate at an influential governmental position	Personal championship characteristics and organization determinants	Relationship of personal and institutionalization connection	Personal championship	Institutional-based incentive in personal championship
MI	13	Able to mobilize formal or informal organization for the AMR policy cause	Positive personal championship characteristics	Relationship of personal and institutionalization connection	Personal championship	Personal and political capacity in personal championship
MI	14	Change of person-in-charge or commitment(positive or negative)	Determinants for policy process as window of opportunity and timing of policy	Relationship of personal and institutionalization connection	Window of opoortunity and timing	Loss of continuity in individual championship
MI	15	Fulfil a task of the office	Positive personal championship characteristics	Relationship of personal and institutionalization connection	Personal championship and role in office	Office role commitment in personal championship
MI	16	Vested personal interest in an established AMR theme or goal by the office or society or peer	Positive personal championship characteristics	Relationship of personal and institutionalization connection	Personal championship	Office, society, personal goal in personal championship
MI	17	Follow up of previous effort in AMR pursuit	Positive personal championship characteristics	Personal championship approaches	Personal championship	Office role commitment in personal championship
MI	18	Extend beyond personal background to accomplish task	Positive personal championship characteristics	Personal championship approaches	Personal championship	Beyond personal obligation in personal championship
MI	19	Confidence from knowledge and sufficient eco-structure/political support to pursue AMR policy	Positive personal championship characteristics	Personal championship approaches	Personal championship and political connect	Political support in personal championship

**MI**	**20**	**Use personal knowledge and capacity to accomplish AMR tasks**		**Positive personal championship characteristics**	**Personal championship approaches**	**Personal championship**	**Use of personal knowledge in championing AMR**
**MI**	**21**	**Level of entry—opinion or assertion limitation at position(low level of in office hierarchy)**		**Negative personal championship characteristics**	**Lack of positional assertion -negative trait to personal championship**	**Personal championship**	**Cannot mobilize positional authority nor championing the cause**
**MI**	**22**	**Methodology or approach of the interviewee that contribute to success of prioritization/adaptation/enactment/implementation**		**Positive personal championship characteristics**	**Personal championship approaches**	**Personal championship**	**Mobilize personal capacity in personal championship**
**MI**	**23**	**Individual/organization cannot overcome hurdle/require government/another entity/another effort to overcome the hurdle/prioritize the AMR agenda or policy.**	**25.1 interviewee sees political commitment/political championship**	**Negative organization dynamics**	**Institutional limitation**	**Shortcoming in institutional policy advocacy**	**Cannot mobilize organizational authority nor championing the cause**
**MI**	**24**	**Economics incentive to place AMR agenda a priority or at policy initiation/adaptation**	**26.1 of opinion AMR mitigation is driven by food export – economic priority 26.2 of opinion AMR mitigation is driven by international/peer pressure**	**Economic determinant**	**Societal factor limits policy**	**Social norm**	**Economics incentive to prioritize AMR policy**
**MI**	**25**	**Personally ensure political process to follow from agenda prioritization to policy process**		**Positive personal championship characteristics**	**Personal championship approaches**	**Personal championship and political connect**	**Mobilize personal capacity to advocate policy**
**MI**	**26**	**NGO intelligence to support AMR policy**		**Societal organisation support**	**Societal factor enables policy**	**Social organization support**	**Mobilize NGO capacity to advocate policy**
**MI**	**27**	**Of opinion that there is insufficient evidence linking AMU and AMR**		**Negative personal championship characteristics**	**Technical evidence fails to persuade AMR policy development**	**Personal championship and personal view**	**Personal view on lack of evidence**

MI	28	Believe AMR will affect personal lives		Personal championship characteristics	Qualitative technical evidence can to persuade policy development	Personal championship and personal belief	Personal belief applied in policy advocacy
MI	29	Of opinion that AMR in the country is a problem of global issue(such as spillover, import etc)		Societal and international view	Global AMR consideration from angle of societal responsibility	Personal view on global spillover	Personal view on international responsibility
MI	30	Of opinion that the AMR national action plan (NAP) has/has not mentioned, facilitate different sectors to implement		Personal championship characteristics	Lack of implementation or factors for implementation	Personal view on implementation shortcomings	Personal view on lack of sectoral persuasion on implementation
MI	31	Of opinion there is insufficient continuous collaboration among sectors(non-outbreak related)		Societal organisation view	Lack of implementation or factors for implementation	Personal belief on collaboration shortcomings	Personal view on lack of sectoral collaboration
MI	32	Of opinion that there is urgency exist internationally, (P31)and spillover to local AMR (import))		Global health view on AMR	Local AMR consideration from angle of global responsibility	Personal belief and local view	Personal view on international responsibility
MI	33	Precautionary principle as personal belief that AMR mitigation will be late when patient outbreak pandemic/endemic occurs	of opinion that Precautionary principle is not sufficient to regulate AGP/Antimicrobial use ban in the country	Personal championship characteristics	Lag between individual awareness of AMR and AMR affecting society	Personal championship with belief on precautionary principle	Personal belief in precautionary principle
MI	34	Of opinion that local effort should be well implemented as a model for neighbours and peers		Societal organisation view	Policy lesson learning	Personal view with view on cross-country learning	Personal view on international policy lesson-learning
MI	35	Continuous (sustainable) policy is an enabler to AMR policy advocacy		Inter-community support on AMR policy and positive feedback view	Policy lesson learning	Personal belief and view on policy sustainability	Personal belief on sustainable policy as a factor to advocate for further policy
MI	36	Bilateral information exchange needed between health service		Community cohort	Opinion for implementation	Personal championship with view on	Personal championship characteritics

		providers and public health providers		positive feedback view		sectoral collaboration	
MI	37	Positive reinforcement or personal gratification in policy-makers to initiation		Positive personal championship characteristics	Personal championship approaches	Personal championship and incentive	Personal championship characteritics
MI	38	Need to balance AMR policy and country's benefit		Societal organisation view and balance of priorities of policies	Local AMR consideration from angle of policy agenda prioritization	Personal championship and personal belief	Personal championship on policy priority responsibility on national interest
MI	39	Of opinion that nature of AMR infections and colonization is different from the tangible/direct causality/virulent diseases (eg HIV)		Scientific evidence and knowledge direction	Technical consideration of AMR	Personal view on AMR	Personal view on nature of AMR
MI	40	Of opinion that expertise opinion is needed to initiate/sustain policy		Technocrat and expert input	Resources to implement AMR policies	Personal view on expertise to sustain policy	Personal view on expertise opinion
MI	41	Of opinion that the policy implementation needs to first address a country/society's basic need.	a. At the moment, not addressed, especially due to limitation in LMIC<br>b. Food security not addressed<br>c. Hospital sanitation not addressed<br>d. Social hygiene concept not addressed<br>e. Patient/AM user's AM stewardship concept not addressed	Societal organisation view and balance of priorities of policies	Local AMR consideration from angle of policy agenda prioritization	Personal view on policy priority	Personal championship on policy priority responsibility on national interest
MI	42	Of opinion in AMR there is a lack of regulation or law-binding regulations		Legislature support to AMR policies and goals	Policy effectiveness in legal-binding considerations	Personal view on legal-binding policies to implement AMR policy	Personal view on legal-binding in AMR policy
MI	43	Of opinion in AMR farm antibiotics therapeutic and prophylactic use needs clearer		Stewardship support to AMR policies and goals	Policy effectiveness in clarity and precision of	Personal view on farm antimicrobial use	Personal view on farm stewardship guidelines and

		definition and continuing education			implementation and stewardship		implementation of AMR policies
MI	44	Top-down policy mismatch with policy-implementation program		Policy implementation improvement	Policy implementation lesson to learn	View on implementation shortcomings	Implementation view
MI	45	Loss of AMR policy implementation and adoption original intention		Policy implementation improvement	Policy implementation lesson to learn	View on implementation shortcomings and original intention	Implementation view
MI	46	Antimicrobial resistance an issue that is confused with, or attention diluted by other associated drug residue issue.		Scientific evidence and knowledge redirection	Issue clarity	Drug residue opinion	Policy prioritization confusion from complexity of evidence
MI	47	Of opinion that one policy for all is impractical		One Health perception and application in AMR	Policy implementation lesson to learn	Personal view on policy diversity	Single-policy approach view
MI	48	Of opinion it is difficult to have representative voice due to large population(farm)		Societal organisation view	Policy implementation lesson to learn	Personal view on policy representativeness and coverage	Personal view on policy representiveness and hurdle in advocacy and implementation in farms
MI	49	Of opinion ethical and moral obligation will not establish policy implementation	Of opinion that moral and ethical obligation requires development	Policy persuasion and AMR policy advocacy view	Policy implementation lesson to learn	Personal view in implementation shortcomings relying on moral obligation	Personal view on moral obligation insufficient to implement policy
MI	50	Lack of evidence to support behavioral change to implement AMR education for patients.		Negative evidence-based for policy advocacy in defined communities	Policy implementation lesson to learn	Personal view on policy evidence in education programs	Personal view on lack of evidence in AM stewardship implementation
MI	51	Believe that providers will change behavior if they are given sufficient knowledge regarding preventive medicine as oppose to treatment.		Scientific evidence and knowledge channeling	Policy implementation lesson to learn	Personal view on knowledge-based behavioral change	Personal view on knowledge-based behavioral change

Table S2. Coding categorized as macro-determinants of AMR policymaking.

Level code	Sub-level code	Open coding - primary variables	Sub-variables-->	Axial Coding-->	Selective coding-->	MA with meso-construct-->	Policy protagonists in policy advocacy and implementation
MA	1	Social influence and norm	1.1 Created social influence from MI<br>1.2 Lack of social norm or urgency<br>1.3 Social fatigue<br>1.3.1 Time-prolonged timeframe/ chronic duration AMR issues span over<br>1.3.2 Difficult to sustain interest<br>1.4 Lack of face to problem	Social influence and timing of policy, duration of policy or closed window of opportunity	Timing and lack of policy window that was originally held out by a small group in society	Personal championship and policy window discourse	Personal championship in social context
MA	2	International organization influence	2.1 International agencies overcome HR shift at government<br>2.2 From country based to prioritize at UN agenda	Policy durability discussion in framework of international agencies and advocacy	International agency appear to hold out policy window longer and wider than local effort.	Institutionalizationed advocacy at international level	Institutionalization
MA	3	Economic influence and pressure	3.1 Economic viability<br>3.2 Economic barrier<br>3.3 Economic incentives for market(Eg Market Entry Rewards)	Institutional or personal persuasion in view of economics trade off perceived in AMR policy development	Policy persuasion and durability	Personal championship characteristics	Personal championship in economic context
MA	4	Resources and capacity availability and limitation		Policy durability discussion in framework of resource availability and contentious resource investment on policy agenda prioritization	Lack of resources for policy implementation	Shortfall in implementation	Implementation discourse

**MA**	**5**	**Legislation ease, hurdles, poltical climate**	**5.1 Government change (in framework/approach/priority) leading to changes in private industry**<br>**5.2 Private industry change (in framework/approach/priority/operando guideline) leading to change in government approach/priority**<br>**5.3 Political support from within government/institution**	**Policy implementation discussion from political stance.**	**Institutional and industrial support challenges**	**Shortfall in implementation**	**Implementation discourse**
**MA**	**6**	**Collaboration or isolation among different office**	**6.1 Collaboration among different sectors**<br>**6.2 Collaboration within sectors**	**Implementation of policy in framework of collaboration among sectors**	**Policy persuasion, durability, and implementation**	**Shortfall in implementation**	**Implementation discourse**
**MA**	**7**	**Consensus, entities and organization within country**	**7.1 Create consensus/consensus among different offices, entities and organization within country**<br>**7.2 Unable to create consensus/consensus among different offices, entities and organization within country**<br>**7.3 Shift of consensus among different offices, entities and organization within country or isolation with other countries and regions**<br>**7.4 Office collaboration a hurdle to AMR policy process/implementation**	**Implementation of policy in framework to seek consensus among sectors**	**Policy persuasion, durability, and implementation difficulties among sectors**	**Shortfall in implementation**	**Implementation discourse**
**MA**	**8**	**Global and regional collaboration**	**8.1 Create consensus/consensus between countries and regions**<br>**8.2 Unable to Create consensus/consensus between countries and regions**<br>**8.3 Help prioritize the issue - globally**<br>**8.4 Between-country synchronization- AMR mitigation as common policy goal**<br>**8.5 Help prioritize issue in a particular country**	**Implementation of policy in framework to seek consensus among countries and regions**	**Implementation difficulties among countries and localities**	**Shortfall in implementation**	**Implementation discourse**
**MA**	**9**	**Professional support or hurdle**	**9.1 Enabler from professional/industrial collaboration or consensus**	**Implementation of policy in**	**Implementation enablers with**	**Shortfall in implementation**	**Implementation discourse**

			9.2 Barrier from lack of professional/industrial collaboration or consensus	framework to seek support from professional bodies	professional support		
MA	10	Cultural, historical, geographical and anthropological contextual influence	10.1 Enabler from cultural, historical, geographical and anthropological contextual influence<br>10.2 Barrier from cultural, historical, geographical and anthropological contextual influence	Cultural and historical factors in policy advocacy	Ethnographic, cultural, and socio-economical inhibitors and enablers of AMR policies	Personal championship and social norm discourse	Personal championship in cultural and social context
MA	11	Disease and resistance patterns	11.1 Enabler from presence or construction of surveillance system<br>11.2 Barrier from lack of surveillance system<br>11.3 Public health as a primary motivation to prioritize AMR<br>11.4 Barrier from lack of surveillance system coordination/sharing among different sectors/industry	Surveillance data as part of technical evidence for policy advocacy and development	Support to policy advocacy and implementation	Shortfall in implementation	Implementation discourse
MA	12	Multiple levels or widespread HR or eco-structure support		Governance establishment as support to AMR policy advocacy	Support to policy advocacy and implementation	Shortfall in implementation	Implementation discourse
MA	13	Brand and recognition of AMR role of leadership		Leadership style and personal championship in an institution	Institutional support	Institutionalizationed advocacy	Institutionalization
MA	14	Timeline management and operational space permissible		Time as frame of reference for AMR policy advocacy	Window of opportunity for policy advocacy in terms of organization	Institutionalizationed advocacy	Institutionalization
MA	15	Knowledge exchange(d) at national and international level		National and international dynamics in AMR policy advocacy	Window of opportunity for policy advocacy in terms of international and national	Institutionalizationed advocacy	Institutionalization

					agencies as a unit		
MA	16	Feasible social atmosphere	lack of feasible atmosphere occurs in some LMIC and communities with contending vested interests	Social influence on AMR policy advocacy	Social determinant	Social norm	Social norm
MA	17	Cultural difference	17.1 Country cultural difference<br>17.2 Individual-to-cultural difference<br>17.3 Government culture<br>17.4 Professional culture<br>17.5 Industrial culture<br>17.6 Public health and health culture	Cultural and historical factors in AMR policy advocacy	Contextual variation across culture, social, and ethnographic in AMR policy development	Social norm	Social norm
MA	18	Quasi-government influence		Governance establishment as support to AMR policy advocacy		Institutionalizationed advocacy	Institutionalization
MA	19	Organizational behavior	19.1 Change of government/organization/industry/institution operational framework<br>19.2 Inertia of government/organization/industry/institution operando framework<br>19.3 Lack of mutual urgency between organisations<br>19.4 Interest/pre-existing preparedness to initiate/adapt policy	Organizational discourse and lack of memory deters AMR policy advocacy continuity especially passing on among agencies	Window of opportunity and organizational challenges determines policy acceptance.	Shortfall of institutionalizationed advocacy	Institutionalization
MA	20	Lack of granularity in policy/program		Policy ambiguity lowers persuasive capacity of policy especially implementing programs with conflict of interests	Policy persuasion, durability, and implementation difficulties among stakeholders	Shortfall in implementation	Implementation discourse
MA	21	Diminished/diminishing financial interest in institution/organization		Financial dis-incentive reduces policy	Financial determinant	Financial conflict of interest	Implementation discourse

				durability in organization			
MA	22	Agency-mismatch		Agency with different policy goals or consideration deters policy advocacy	Institutional and industrial support challenges	Shortfall in institutionalizationed advocacy	Institutionalization
MA	23	Cooperate responsibilities	23.1 See AM responsibility as social good<br>23.2 Cooperate culture<br>23.3 Cooperate leadership	Social good and cooperate culture affects policy advocacy	Cooperate culture affects policy advocacy	Social norm	Social norm
MA	24	Individual capacity captured into organization		Individual championship and institutional alignment is crucial	Personal championship and institutional culture	Personal championship and policy window alignment	Personal championship and institutional alignment
MA	25	Government interest enabler	25.1 perceived benefit to be leader in field<br>25.2 perceived benefit to food chain safety<br>25.3 perceived benefit to social norm change	Political interest in government as a unit	Political determinant	Political persuasion	Institutionalization challenges
MA	26	International country-based enabler		Policymaking among international agencies and national offices need to coincide in terms of entrepreneurs communication	Personal championship and international institutional culture	Personal championship and institutionalized policy window alignment	Personal championship and international institutional advocacy
MA	27	Media as an enabler		Media influences consumer especially in food safety related to AMR	Policy implementation challenges in persuading public to pay for "antibiotic-free" food products	Social norm	Social norm

MA	28	Delayed real-time report in surveillance system		Surveillance data as part of technical evidence for policy advocacy and development	Deterrant of surveillance system as an AMR investment	Shortfall in institutionalized resources	Institutionalization
MA	29	Delayed AMR process due to administrative or other resources or infrastructure	29.1 Administrative process<br>29.2 Lack of knowledge<br>29.3 Lack of resources<br>29.4 Lack of public education<br>29.5 Lack of regulation	Implementation deterrant	Implementation deterrant	Shortfall of institutionalizationed advocacy	Institutionalization
MA	30	Need for pharmaceutical research		Innovation challenges	Incentive and resource limitation	Shortfall in institutionalized resources	Institutionalization
MA	31	Longer term planning for NAP needed		Implementation challenge result from planning gaps	Policy amnesia and durability challenge		Institutionalization challenges
MA	32	Specialist engagement in AMR policies needed		Expertise' role in AMR evidence interpretation for policy advocacy and implementation	Personal championship and institutional culture		Personal championship and institutional alignment
MA	33	International collaboration and implementation at international level essential		International institutional persuasion cannot overcome local policy amnesia	Institutional and local policy durability challenge	Shortfall in institutionalizationed advocacy	Institutionalization challenges
MA	34	Unique country background in general		Ethnographic and cultural differences affects policy advocacy consideration	Policy development needs cultural and ethnic contextual consideration	Shortfall in social norm	Social norm
MA	35	Championship – group championship		Advocacy coalition consideration	Advocacy coalition	Coalition advocacy	Coalition advocacy

MA	36	Leadership is important		Personal championship and political alignment	Policy entrepreneurship	Personal championship and policy window alignment	Personal championship in political context
MA	37	Cultural change over period of time		Timing and cultural determinant	Timing and communication approach influenced by local culture and etiquette	Personal championship and policy window alignment	Personal championship in policy window and timing
MA	38	Nation/country-based perspective and economic/GDP improvement	Nation-based size—difficulty due to large size of country/ease due to small size of country/population	AMR policy advocacy reach limitation due to diverse demographics or large population and social characteristics	National characteristics influence AMR policy advocacy outcome	Shortfall in implementation	Implementation discourse
MA	39	Country based varying approach to AGP (banning, restricted use, permit to use)		Farming characteristics affect AMR policy effectiveness	Country with different farming culture and infrastructure influences AMR policy advocacy	Social norm and shortfall in implementation	Social norm
MA	40	Lack of surveillance data and situation analysis-therefore lack of implementation of policies		Surveillance data as part of technical evidence for policy advocacy and development	Implementation challenge from lack of evidence	Shortfall in implementation	Implementation discourse
MA	41	Food security an issue	Better lifestyle in Low- to middle-incomeI countries lead to an increase in AMU in farms to raise food-producing animals	Controversial AMU regulation policies in farm animals and animal	Lack of persuasion or high resistance in AMU policy at implementation stage	Social norm and shortfall in implementation	Social norm

			protein production			
MA	42	Country-based-cultural and political choice on international peer pressure eg close door policy	Pros and cons relying on cross-country policy learning	Cultural and political sensitivity across countries affects AMR policy advocacy approach	Inter-national norm	International support
MA	43	Country-based consumer culture difference	Consumer culture	Implementation challenge from sufficient or lack of consumer support	Social norm and shortfall in implementation	Social norm
MA	44	Country-based media involvement	Media influences consumer especially in food safety related to AMR	Media influence	Media and implementation characteristics	Implementation characteristics
MA	45	Country-based improvement in public health representation in society	Governance establishment as support to AMR policy advocacy	Public health governance and baseline affects how much or little AMR policy advocacy can be established	Institutionalizationed advocacy	Institutionalization
MA	46	Country level financial support to improve farm conditions, hygiene and technology (a financial incentive for the nation with export commodity)	Financial status of food-animal production as an industry affects willingness to accept AMU regulation policy	AMR policy window of opportunity influenced by baseline well-being of food-producing animal farming industry	Implementation consideration	Implementation characteristics

Table S3. Interview themes, sub-themes, quote identifier, and quotes.

Main themes	Sub-themes	Quotes Numbers	Interview quotes
2.1 Individual championship is pivotal but insufficient in the AMR policymaking arena	2.1a Difficult policy implementation	2.1.1	"If I had chance to redo- the whole process, I would have included policy implementation in the whole push for policies among countries. The implementation has been stalled. We have suc-cessfully pushed for AMR policies in some countries but the implementation in the country has not been successful in many." (UK)
		2.1.2	"…But I also think the system did not quite put things together into a longer term,…sort of UK's own plan but not enough how we are going to the world in driving this whole thing through. So to me there is two phases actually. I think it was easier to do the first bit than people realized, but harder to do the second bit sort of putting stuff in practice." (UK)
		2.1.3	"..it is a combination of lack of understanding, technically complicated and who holds the power. And we all know the finance ministries hold the power and they have not bought this yet and health ministers hold no power at all…and the Agriculture ministers not wanting to move into this because it threatens their food chains and their private sectors are saying, "no don't go there." (UK)
		2.1.4	"..we do not have this stable basis of political support and we do not have the public mandate necessarily to achieve that…that becomes particular important once we start heading into kind of more difficult questions around how to fund things." (US)
	2.1b AMR policy protagonists who were allowed to advice policy at stages of policy ini-tiation and formulation helped converge public, professional, and policy perspectives.	2.1.5	"..I think the key driver, to my mind was [the] Chief Medical Officer,…as I saw it, the opening of that (AMR) conversation and escalating it to a government, cross-government level and a political priority,…." To move AMR at the international space, "..a couple of strands that was domestic conversation…, and the backing of government, for it (AMR prioritization) to be effective,…the idea of taking some action on this at global level, moving out of that technical, medical space and bring it into the political arena." (UK)
		2.1.6	"…we have levels of connectivity that supersede the political sphere…we had very very strong high level White House support during the Obama administration that lessened during Trump administration. Secretary is bound and determined he wants to bring..economic incentive to help antibiotic discovering commercialization before he leaves. So, you know, the political are very very involved. Under that there is a level of senior executive service like myself who..going to say 7 out of 13 originals were part of the effort in 2014, and still in place. And then you have

			staff level engagement that is literally day-to-day talking with their counterparts. But that has been built over time." (US)
		2.1.7	" …in China, it's not like this. I know the United Kingdom, in the United States, there is a presidential Department committee doing this. In China, there is not such a high steering committee, … a cross-departmental institution that is higher than the Department, it may not be realistic in the short term, first of all, the understanding of AMR issue, and for the management department to understands, may also need to continue to educate them, …understanding them, this is very important. AND "…my personal comment on NAP is it should involve food safety, the preparation (of NAP) is very short…the food safety department was not involved." (China)
		2.1.8	"…it's not that easy to push the establishment of a higher level of interdepartmental (AMR) coordination"(China)
		2.1.9	"…main role of the institute is to provide facts and information and knowledge about the AMR problem in Norway. And we are also an advisor for the government and also for the health sector, especially the human health sector. The institute is also a research institute, that has a role in establishing research projects and establishing networks with other institutions, universities, high schools and also networks across with the research groups in other countries." (Norway)
2.2 Policy institutionalization facilitates AMR policy prioritization and implementation	2.2a Institutionalisation plays key role	2.2.1	"…continuation is most important. We need people to ensure continuation. I think it is important to create an environment and a mechanism on site, where people can be involved in AMR, and to foster human development…" (Japan)
		2.2.2	"…the change of administration we definitely saw a deprioritization…decrease in budget and staff size. I assume when another administration comes along that may change. While the CDC (human) and USDA (foodborne sector) pick up the slack, they focused the attention on people who are specialists within the environment from academia and the private sector who could help at least articulate in the form of report…and major questions that need to be addressed to help with policymaking…" (US)
		2.2.3	"…evolving..it has been hard to mobilize the interest within the White House that we used to have. Part of that is because the changeover in staff, no one actually was assigned to the AMR portfolio. And so when you do not have somebody who was covering it day in and day out and actually pushing the policy agenda it reverted to the departments and agencies doing all the work. And that is, it is just a different type of process…" (US)
2.3 Free markets play an ambivalent role while social norm a driver in AMR policymaking		2.3.1	"..if (AMU in animals) are tightened too much, animal husbandry will decline, and large quantities of foreign

		**products will be on the market in Japan, whose food self-sufficiency rate is already low." (Japan)**
	**2.3.2**	**"For basic public education, "…it has to be among younger generation,…I channeled private funding to facilitate public awareness program." (China)**
	**2.3.3**	**Across countries, economic incentive link or delink in medical insurance infrastructure, reimbursement policies, and hospital income generation were used to change social behavior and implement AMR stewardship programs. Interviewees from pharmaceutical leadership opined financial dis-incentives, however, have relegated pharmaceutical innovations efforts.**
	**2.3.4**	**"So you are never going to come up when they (president) are campaigning. So to get this political will thing to work and get real champions its… money. Money talks here and when reimbursement policies really come down to that reinforce these policies, then there will be change. Secondly, are the stories that can be told. Particular when influential people have family members or loved one or even themselves have been impacted by these diseases." (US)**
	**2.3.5**	**"I think the major incentive in the United States comes from the payer. That is insurance company. Because the hospitals and providers to be reimbursed…Medicare rule that reimbursement must have antimicrobial use policies in place". (US)**
	**2.3.6**	**"… public education needs to be supported with change of mentality on intravenous drip and antibiotics use on treating fever…to stop linking income with medicine use is the most important." and "…Medicine charges are a large piece of medical income…doctors need to sustain services to improve medical services." (China)**
	**2.3.7**	**"…pharma innovation.. this kind of conversation has been going on and not really getting into the details and implementation,..they are now hitting point where investor confidence is tailing off and they are running out of money.." (UK)**
